# Impact of Negative Fluid Balance on Mortality and Outcome of Patients with Confirmed COVID-19

**DOI:** 10.1155/2023/6957341

**Published:** 2023-06-05

**Authors:** Seyed Parsa Eftekhar, Mahdi Sepidarkish, Parviz Amri Maleh, Iraj Jafaripour, Mohammad Taghi Hedayati, Kamyar Amin, Roghayeh Pourkia, Saeid Abroutan, Mehrdad Saravi, Farzad Jalali, Mahmoud Sadeghi Haddad Zavareh, Naghmeh Ziaie

**Affiliations:** ^1^School of Medicine, Babol University of Medical Sciences, Babol, Iran; ^2^Department of Biostatistics and Epidemiology, School of Public Health, Babol University of Medical Sciences, Babol, Iran; ^3^Department of Anesthesiology, Babol University of Medical Sciences, Babol, Iran; ^4^Department of Cardiology, Babol University of Medical Sciences, Babol, Iran; ^5^Infectious Diseases and Tropical Medicine Research Center, Health Research Institute, Babol University of Medical Sciences, Babol, Iran

## Abstract

**Purpose:**

Maintaining the proper fluid balance is a fundamental step in the management of hospitalized patients. The current study evaluated the impact of negative fluid balance on outcomes of patients with confirmed COVID-19.

**Methods:**

We considered the negative fluid balance as a higher output fluid compared to the input fluid. The fluid balance was categorized into four groups (group 4: −850 to −500 ml/day; group 3: −499 to −200 ml/day, group 2: −199 to 0 ml/day, and group 1 : 1 to 1000 ml/day) and included ordinally in the model. The outcomes were all-cause mortality, length of hospitalization, and improvement in oxygen saturation.

**Results:**

The fluid balance differed significantly among nonsurvivors and survivors (MD: −317.93, 95% CI: −410.21, −225.69, and *p* < 0.001). After adjusting for potential confounders, there was a significantly lower frequency of mortality in patients with negative fluid balance compared to the controls (aRR: 0.69, 95% CI: 0.57, 0.84, and *p* < 0.001). Similarly, the length of hospitalization was significantly shorter in the negative fluid balance group in comparison to the control group (aMD: −1.01, 95% CI: −1.74, −0.28, and *p*=0.006).

**Conclusion:**

We determined that the negative fluid balance was associated with favorable outcomes in COVID-19 patients. The negative fluid balance was associated with the reduced mortality rate and length of hospitalization as well as improvement in oxygen saturation. Moreover, the NT-proBNP >781 pg/mL and fluid balance >−430 mL might be the predictors for positive fluid balance and mortality, respectively.

## 1. Introduction

In December 2019, severe acute respiratory syndrome coronavirus 2 (SARS-CoV-2) emerged in Wuhan, China, and spread rapidly worldwide. Death through COVID-19 could result from acute respiratory distress syndrome (ARDS) and respiratory failure, cytokine storm and subsequent multiorgan failure, and cardiovascular events. Although respiratory failure is one of the major causes of death among COVID-19 patients, the high mortality rates among the intensive care unit (ICU)-admitted patients suggest the multifactorial nature of COVID-19 [1]. Moreover, comorbidities increase the risk of mortality in COVID-19 patients. Twenty-six percent of deaths have an underlying disease, and sixty-five percent have cardiovascular disorders. Patients with cardiovascular disease are at a higher risk of severe disease development [2].

Increased production of inflammatory cytokines and bradykinin contributes to the increased permeability of pulmonary vessels and fluid accumulation in lung interstitial and alveolar space. This process leads to pulmonary hypertension and exerts an additional overload on the right ventricle [3]. It is worth mentioning that cardiomyopathy and heart failure due to COVID-19 could be associated with fluid overload [4]. Brain natriuretic peptide (BNP) and N-terminal (NT)-pro hormone BNP (NT-proBNP) are quantitative markers representing cardiac hemodynamic stress and have an important role in diagnosis and following up the patients with heart failure [5]. Acute renal failure (ARF) is another adverse event, especially in critically ill patients [6]. The ARF leads to fluid overload and electrolyte imbalance, which might deteriorate the pulmonary edema [7]. In the same line, ARDS is frequently associated with shock, and proper fluid management contributes to adequate tissue perfusion while avoiding positive fluid balance and tissue edema is necessary [8]. Moreover, it has been suggested that angiotensin-converting enzyme II (ACE-II) is higher in COVID-19 patients compared to the healthy population, which upregulates aldosterone and contributes to sodium and water retention [9].

Studies have showed that early resuscitation with intravenous fluids is essential for the improvement of hemodynamic stability, tissue perfusion, and mortality reduction in ICU admitted patients. Moreover, studies showed that negative fluid balance might be associated with promising outcomes in ARF, heart failure, and ARDS in non-COVID-19 patients. However, the negative fluid balance efficacy in COVID-19 patients is not well known. Additionally, it has been suggested that positive fluid balance could deteriorate the patients' general condition by affecting the renal, cardiac, and pulmonary functions. The fluid overload is associated with pulmonary and peripheral edema, increased chance of respiratory failure, delayed weaning from mechanical ventilation, and poor prognosis [8]. We aimed to characterize the association between negative fluid balance and mortality, length of hospitalization, and oxygen saturation in COVID-19 confirmed patients. We also hypothesized that the level of NT-proBNP might be a potential predictor for furosemide administration.

## 2. Methods

### 2.1. Design and Setting

This retrospective cohort study was conducted in two university hospitals in the Babol city, north of Iran, from July 2020 to May 2021. This study has been approved by the Ethics Committee of Babol University of Medical Sciences (the ethical code: IR.MUBABOL.REC.1399.425). Before enrolment, written informed consent was obtained from study participants.

### 2.2. Patient Characteristics

All adult patients (both general ward and ICU) in the two university hospitals were screened for inclusion and exclusion criteria. We defined the cohort as hospitalized patients with positive reverse transcriptase-polymerase chain reaction (RT-PCR) for SARS-CoV-2. An infectious disease specialist verified the positive RT-PCR tests and confirmed the infection with clinical symptoms and computed tomography (CT) scan finding (ground glass opacification). Inclusion criteria included (1) age ≥18 years old and (2) normal and acceptable range of serum electrolytes. The exclusion criteria were (1) age <18 years old; (2) pregnancy; (3) development of major arrhythmic events, including sudden cardiac death (SCD), sudden cardiac arrest (SCA), ventricular fibrillation (VF), and supraventricular tachycardia (sVT); and (4) changing the treatment protocol such as the application of plasmapheresis or adding a new antiviral agent. We defined critical illness as the presence of respiratory failure, septic shock, and/or multiorgan failure. The treatment protocol was remdesivir, dexamethasone, and heparin. Moreover, 166 patients received invasive ventilation and six patients needed dialysis.

### 2.3. Data Collection at Recruitment

We retrieved patients' characteristics such as demographics, clinical features, comorbidities, and laboratory findings on admission from medical records. A trained team of researchers reviewed and retrieved data for each patient.

### 2.4. Fluid Status Assessment

The daily net fluid balance was calculated by subtracting daily fluid output from daily fluid input. Fluid input included oral, enteral, and intravenous fluid. We used calibrated containers for measuring the oral and enteral fluid volume. Fluid output included urine and fluid loss from drains and tubes. Urine volumes were collected and measured via Foley catheter and urine bag. Fluid input and output were calculated (milliliters per day) and charted every 24 hours. We considered the negative fluid balance as a higher output fluid compared to the input fluid. We applied oral and intravenous fluid restriction and administered furosemide (loop diuretic). We adjusted the fluid balance based on the presence of congestion, vital signs, clinical findings, and laboratory findings such as the creatinine level. After determining the proper fluid balance in each patient, we maintained the established amount during the hospitalization. However, we overlooked minor changes in fluid balance during hospitalization.

### 2.5. Outcomes and Definition

The primary outcome was all-cause mortality. The length of hospitalization and improvement in O_2_ saturation were selected as the key secondary endpoints.

### 2.6. Statistical Analysis

All statistical analyses were performed with STATA 16 (Stata Corp, College Station, TX, USA) and GraphPad Prism 9 software (GraphPad Software Inc., La Jolla, CA, USA). The baseline characteristics were compared between the two groups using the independent sample *t*-test for continuous data and a chi-square test for categorical data. Prior to statistical comparisons, all data were tested for normal distribution by the Kolmogorov–Smirnov test and visually by the Q-Q plot. We used receiver operating characteristic (ROC) curves to identify the classification threshold of NT-proBNP and fluid balance. We reported diagnostic indices such as area under the curve (AUC), sensitivity, specificity, and likelihood ratio (LR). We estimated the risk ratios (RRs) and 95% confidence intervals (CIs) for mortality using a negative log-binomial regression model. The linear mixed effects models were fit to assess changes from the baseline within the groups and differences of those changes between groups with respect to oxygen saturation during hospitalization and the length of hospital stay. Fluid balance was categorized into four groups and included ordinally in the model (Tables 1 and 2). The magnitude of the effect is presented as the adjusted mean difference (aMD) and its 95% confidence interval. The independent variables included in the model were fluid balance, age, gender, prior heart failure, chronic kidney disease, and critical illness. The significance was defined as *p* < 0.05.

## 3. Results

The recruitment of patients was commenced in July 2020 and ended in May 2021. During eleven months, a total number of 315 patients were evaluated and 294 patients were included (according to inclusion and exclusion criteria). The demographic and baseline characteristics of the patients are summarized in Table 3. One hundred forty-two patients (48.29%) were female, the mean age of the patients was 64.33 ± 16.38 years (median: 66, IQR: 77 − 56 years), and the proportion of patients with critical illness was 63.6%. Two hundred forty patients (81.63%) had at least one pre-existing condition. The mean age was 66.77 years (SD = 16.56) in deceased patients and 63.01 (SD = 16.21) in the survivors (*p*=0.064). Furthermore, deceased patients had almost the same frequency of pre-existing medical problems (82/101, 81.18%) compared with the control group (158/193, 81.86%) (*p*=0.886). The laboratory results showed that interleukin-6 (IL-6) (MD: 55.4, 95% CI: 8.71, 102.09; *p*=0.020) and c-reactive protein (CRP) (MD: 26.14, 95% CI: 2.71, 49.57; *p*=0.029) were significantly higher in deceased patients compared to the survivors. No statistically significant differences existed in the creatinine levels on the day of hospital admission and before hospital discharge or death (Change difference: 0.06, 95% CI: −0.1, 0.1; *p*=0.943). The mean ± (SD) of NT-proBNP in all patients at the time of admission was 8254 ± (10061.2) pg/mL. Moreover, we measured the NT-proBNP before discharging survived patients. The NT-proBNP was significantly lower in survived patients before discharge compared to the time of admission (MD: 4758.5, 95% CI: 3725.2, 5791.7; *p* < 0.001).

**Figure 1:**
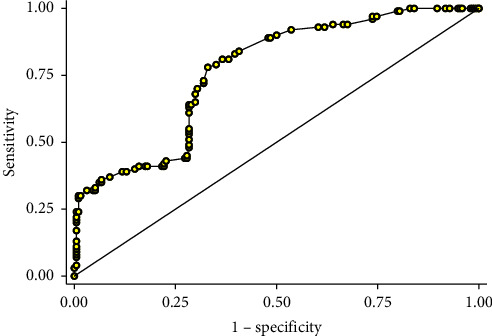
The area under the ROC curve for fluid balance as a classifier of mortality was 76.5 (95% CI: 71.2, 81.2). A fluid balance level of >−430 mL was associated with mortality, with a sensitivity of 68.8% (95% CI: 61.5, 74.5), a specificity of 73.5% (95% CI: 63.2, 81.4), a positive likelihood ratio (LR) of 2.52 (95% CI: 1.81, 3.53), and a negative LR of 0.43 (95% CI: 0.34, 0.55).

**Figure 2:**
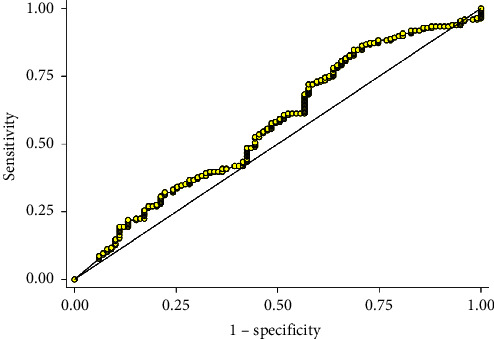
The area under the ROC curve for NT-proBNP levels as a predictor of positive fluid balance was 63.2 (95% CI: 55.6, 70.4). A NT-proBNP level of >781 pg/mL was associated with positive fluid balance, with a sensitivity of 55.8% (95% CI: 41.3, 69.5), a specificity of 70.2% (95% CI: 64.1, 75.9), a positive likelihood ratio (LR) of 1.87 (95% CI: 1.37, 2.56), and a negative LR of 0.63 (95% CI: 0.45, 0.86).

One hundred ninety-six patients (66.66%) achieved negative fluid balance. In the two hundred ninety-four patients included in this study, the mean ± (SD) of fluid balance was −268.61 ± (408.99) mL (Median: −250, IQR: −550, 240 mL) and differed significantly among nonsurvivors (−58.80 ± 414.57) (Median: 150, IQR: −100, 350 mL) and survivors (−376.75 ± 362.24) (Median: −390, IQR: −570, 100 mL), (MD: −317.93, 95% CI: −410.21, −225.69, and *p* < 0.001) (Table 3). The area under the curve (AUC) for fluid balance as a classifier of mortality was 76.5 (95% CI: 71.2, 81.2) (Figure 1). A fluid balance level of >−430 mL was associated with mortality, with a sensitivity of 68.8% (95% CI: 61.5, 74.5), a specificity of 73.5% (95% CI: 63.2, 81.4), a positive likelihood ratio (LR) of 2.52 (95% CI: 1.81, 3.53), and a negative LR of 0.43 (95% CI: 0.34, 0.55) (Figure 1). The median (IQR) NT-proBNP level was 4,314 (10,749 to 1,218) pg/mL. The area under the ROC curve for NT-proBNP levels as a predictor of positive fluid balance was 63.2 (95% CI: 55.6, 70.4) (Figure 2). An NT-proBNP level of >781 pg/mL was associated with positive fluid balance, with a sensitivity of 55.8% (95% CI: 41.3, 69.5), a specificity of 70.2% (95% CI: 64.1, 75.9), a positive LR of 1.87 (95% CI: 1.37, 2.56), and a negative LR of 0.63 (95% CI: 0.45, 0.86).

The unadjusted result showed a significantly lower frequency of mortality in patients with negative fluid balance compared to the controls (RR: 0.31, 95% CI: 0.19, 0.51, and *p* < 0.001). Moreover, after adjusting for potential confounders, there was a significantly lower frequency of mortality in patients with negative fluid balance compared to the controls (aRR: 0.69, 95% CI: 0.57, 0.84, and *p* < 0.001).

After the enrolment day, the length of hospitalization was significantly shorter in the negative fluid balance group compared to the positive fluid balance group (MD: −0.73, 95% CI: −1.45, −0.01, and *p*=0.046). Similarly, after adjusting for potential confounders, the length of hospitalization was significantly shorter in the negative fluid balance group in comparison to the controls (aMD: −1.01, 95% CI: −1.74, −0.28, and *p*=0.006) (Table 2).

**Figure 3:**
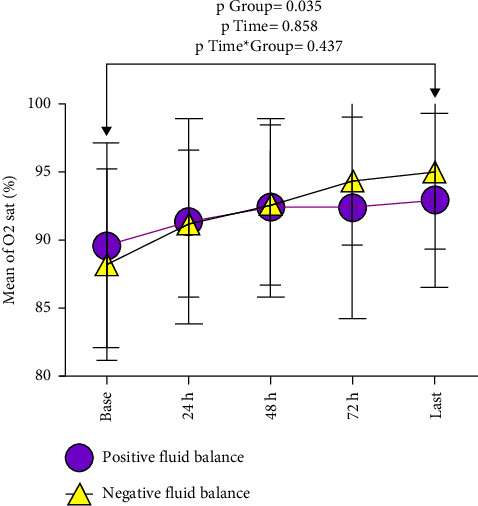
The mean of O_2_ saturation for patients with negative and positive fluid balance at each time-point. All two groups showed a significant increment in O_2_ saturation over time. However, during hospitalization, the negative fluid balance group showed a significantly greater increment compared to the positive fluid balance group (aMD: 1.10, 95% CI: 0.41, 1.80, and *p*=0.035).

Figure 3 shows the mean of O_2_ saturation for patients with negative and positive fluid balance at each time-point. Both groups showed a significant increment in oxygen saturation over time. However, during the hospitalization, the negative fluid balance group showed a significantly greater increment compared to the positive fluid balance group (aMD: 1.10, 95% CI: 0.41, 1.80, and *p*=0.035).

## 4. Discussion

We showed that negative fluid balance was associated with a decreased mortality rate, even after adjusting for the potential confounders such as the presence of critical illness. We categorized the fluid balance into four groups and included them ordinally in the model. For every one level increase in the fluid balance category, there is a decrease of ∼30% in the mortality rate (e.g., −850 to −500 ml group had ∼30% lower mortality rate compared to −499 to −200 ml group). The same model was applied for the length of hospitalization, and as is shown in Table 2, for every one level increase in the fluid balance group, there is a decrease of one day in the length of hospitalization. These findings, alongside the improvement in oxygen saturation, provide a promising approach for managing COVID-19 in critically ill patients.

The ventricle pressure-volume relationship is curvilinear, and the atrial pressure increases rapidly as the ventricle reaches the plateau of the Frank–Starling curve. Elevated atrial pressure contributes to pulmonary and venous hydrostatic pressure overload, associated with the intravascular fluid shift to interstitial space and disturbed capillary blood flow and oxygen saturation [10]. The above effect is more remarkable in patients with COVID-19 due to the active inflammatory process in the lungs, which disturbs the lungs' function. Furthermore, many patients with COVID-19 develop ARDS, which contributes to increased capillary permeability and pulmonary edema and puts patients in more danger against resuscitation-induced lung congestion [11]. Moreover, following the aggressive fluid resuscitation, increased atrial pressure contributes to elevated venous pressure in abdominal organs (retrogradely), including the liver, kidneys, and intestine. The kidneys are susceptible to increased interstitial pressure and tubular compression due to renal intracapsular tamponade, which intensifies adverse events [11].

Both negative and positive fluid balance groups had low oxygen saturation at the beginning of admission. However, at the time of admission, the negative fluid balance had lower oxygen saturation compared to the positive fluid balance group. Pulmonary edema secondary to ARDS or heart failure could be the possible reason for low oxygen saturation in patients [12, 13]. Moreover, the negative fluid balance group showed a significantly higher oxygen increment compared to the positive fluid balance group. This finding suggests that negative fluid balance might apply beneficial effects by reducing the pulmonary edema. In the same line, Kevorkian et al. reported that the combination of corticosteroids and furosemide improves the patients' outcomes, especially in aged patients with comorbidities and who are at a risk of pulmonary edema (BNP >100 ng/mL). However, they evaluated the efficacy of furosemide in noncritically ill COVID-19 patients [14].

The present study showed that males were associated with higher mortality rates compared to females. This finding is consistent with other studies. The studies suggested that COVID-19 has higher severity and mortality in males compared to females, especially in ICU-admitted patients [15, 16]. This finding has been reported in several countries [17]. Moreover, a meta-analysis, including 120 studies and 125,546 participants, showed that severity and mortality are higher among males [18]. Several mechanisms have been suggested for the abovementioned findings, including sex-based differences in the expression of ACE2 receptor and transmembrane protease serine 2 (TMPRSS2) and sex-based differences in immunological response [19]. However, the beneficial effects of negative fluid balance remained statistically significant after adjusting the gender as a confounder.

Hypoxemic respiratory failure (ARDS) is the most common cause of admission to the ICU and is responsible for more than 80% of deaths [2]. It has been shown that the elevated level of cytokines such as IL-6 is associated with poor prognosis and an increased risk of ARDS [20]. In the same line, in the present study, IL-6 was significantly higher in deceased patients compared to survived patients and might imply the role of ARDS in disease prognosis. Hemodynamic instability frequently occurs in patients with ARDS, especially in ICU [21, 22]. More than 60% of patients with ARDS develop hemodynamic instability, and catecholamine administration is necessary in 65% of cases [21, 23]. Similar to other conditions with hemodynamic instability, it might be thought that aggressive fluid administration should be the mainstay of the treatment strategy. In several studies, it has been noted that aggressive fluid resuscitation is associated with poor outcomes in critically ill non-COVID-19 patients [24–26]. Nevertheless, increased intravascular volume next to fluid resuscitation contributes to transient improvement in hemodynamic parameters such as cardiac output [27].

It has been shown that AKI is a common (5.1% to 29%) complication in critically ill COVID-19 patients, associated with high mortality, especially in ICU-admitted patients [28]. Moreover, critically ill children with COVID-19 could develop AKI, and elevated IL-6 is a common finding in these patients, emphasizing the role of cytokine storm as an underlying cause of acute renal injury [29]. High-levels of IL-6 and CRP, as we saw in this study, might be a predictor of renal injury in critically ill patients [30]. Fluid overload is an independent risk factor for ICU mortality, and it necessitates preventing fluid overload in COVID-19 patients, especially in critically ill patients with impaired renal function [31, 32]. In the present study, no statistically significant differences were observed in creatinine levels between the survivor and nonsurvivor groups on the day of hospital admission and before hospital discharge or death. However, the creatinine levels were higher than the healthy population, which might indicate the COVID-19-related AKI.

Several studies evaluated the impact of loop diuretic administration and negative fluid balance on ICU-admitted patients and conditions such as AKI. Moreover, there is no consistency between these studies. Some suggested beneficial effects [33, 34], while others noted harmful or no effects [35–37]. Our results are in the same line with the study of Francisco Santos et al. [9], which evaluated the role of negative fluid balance in COVID-19 patients' outcomes. However, we have a much bigger sample size (294 vs. 20). Moreover, we adjusted the critical illness as an important confounder and determined the role of NT-proBNP level as a predictive tool for loop diuretic administration.

In addition to respiratory failure and shock, critically ill patients with COVID-19 might manifest heart failure [13]. Studies showed that an elevated level of NT-proBNP is strongly associated with a higher mortality rate in COVID-19 patients [38, 39]. The sensitivity analysis suggested that furosemide administration is helpful in the concentration of NT-proBNP >781 pg/mL. In other words, NT-proBNP >781 pg/mL is associated with positive fluid balance. Moreover, the findings showed that the patients who had survived had a statistically significant reduction in the level of NT-proBNP. This finding might be due to improved cardiac function through reduced volume overload.

Physicians should be vigilant during the aggressive fluid administration in COVID-19 patients who have declined cardiac output secondary to cytokine storm, heart failure, or other causes that has no beneficial effects, and overload might lead to adverse results due to pulmonary infiltration that contributes to diminished respiratory function.

As our study showed, the negative fluid balance in critically ill patients was associated with better outcomes. Murphy et al. noted that the administration of adequate and early resuscitation fluid therapy besides conservative late fluid therapy, in patients with acute lung injury, is associated with better outcomes compared to the patients with only one of these approaches or none of the two [40]. Therefore, a dynamic approach and optimal timing (negative fluid balance secondary to early and adequate resuscitation) are the keys to fluid management of patients with hemodynamic instability.

We eliminated an important confounder, critical illness, which was not adjusted in similar studies. Additionally, we used a large database with high granularity. However, the current study has several limitations. First, this is a retrospective study, and we cannot eliminate the residual confounding due to unobserved factors. Furthermore, we did not include insensible perspiration in the fluid balance calculation. In addition, we did not evaluate the NT-proBNP levels in deceased patients. Moreover, we are unable to derive a definite mechanism for the beneficial effects of diuretics and negative fluid balance. Accordingly, we highly recommend prospective studies in this context. It is essential to explore whether more precise fluid management through invasive methods such as central venous pressure measurement or pulmonary arterial catheterization or noninvasive methods such as bioimpedance spectroscopy or echocardiography is necessary or not in a particular subset of patients, including those with underlying heart or renal failure.

In summary, we determined that negative fluid balance was associated with favorable outcomes in COVID-19 patients. The increase in oxygen saturation and decrease in mortality and length of hospitalization may be due to the improvement in the cardiovascular and pulmonary function and tissue perfusion. Although we cannot securely determine that negative fluid balance is beneficial, it is highly likely that negative fluid balance affords COVID-19 patients' material benefit. In the absence of compelling contradictory data from a randomized, blinded clinical trial, we should encourage maintaining negative fluid balance in COVID-19 patients, especially critically ill patients and higher NT-proBNP levels.

## Figures and Tables

**Table 1:** Association between minimal sufficient adjustment sets with mortality among COVID-19 patients.

	Crude RR (95% CI)	*p* value	Adjusted RR (95% CI)^†^	*p* value^*∗∗*^
Fluid balance^*∗*^	0.31 (0.19, 0.51)	<0.001	**0.69 (0.57, 0.84)**	**<0.001**
Prior heart failure	1.12 (0.63, 1.90)	0.735	1.13 (0.62, 2.11)	0.678
Critical illness	5.22 (2.61, 10.47)	<0.001	**5.17 (2.51, 10.61)**	**<0.001**
Chronic kidney disease	0.72 (0.23, 2.21)	0.572	0.91 (0.23, 3.51)	0.887
Gender	1.53 (1.03, 2.57)	0.037	1.01 (0.93, 2.56)	0.101
Age	1.01 (0.99, 1.02)	0.180	1.01 (0.99, 1.02)	0.102

^†^Risk ratio estimated directly from negative log-binomial regression. The final multivariable models were adjusted for the aforementioned risk factors in the table. ^*∗*^Fluid balance was categorized into four groups (group 4: −850 to −500 ml/day, group 3: −499 to −200 ml/day, group 2: −199 to 0 ml/day, and group 1 : 1 to 1000 ml/day (positive fluid balance)). Fluid balance was included ordinally in the model. ^*∗∗*^Significant at *p* value <0.05. The fluid balance and critical illness are significant (*p* value < 0.05).

**Table 2:** Association between minimal sufficient adjustment sets with the length of hospitalization among COVID-19 patients.

	Crude MD (95% CI)	*p* value	Adjusted MD (95% CI)^†^	*p* value^*∗∗*^
Fluid balance^*∗*^	−0.73 (−1.45, −0.01)	0.046	**−1.01 (−1.74, −0.28)**	**0.006**
Prior heart failure	−1.87 (−4.21, 0.46)	0.117	−2.02 (−4.47, 0.41)	0.104
Critical illness	4.51 (2.61, 6.40)	<0.001	**4.82 (2.84, 6.81)**	**<0.001**
Chronic kidney disease	−1.93 (−6.10, 2.23)	0.362	−0.87 (−5.31, 3.57)	0.701
Gender	−0.40 (−2.25, 1.44)	0.669	−0.87 (−2.78, 1.02)	0.367
Age	0.01 (−0.04, 0.07)	0.525	0.02 (−0.03, 0.08)	0.399

^†^Mean difference estimated directly from the mixed-effects linear model. The final multivariable models were adjusted for the aforementioned risk factors in the table. ^*∗*^Fluid balance was categorized into four groups (group 4: −850 to −500 ml/day, group 3: −499 to −200 ml/day, group 2: −199 to 0 ml/day, and group 1 : 1 to 1000 ml/day (positive fluid balance)). Fluid balance was included ordinally in the model. ^*∗∗*^Significant at *p* value <0.05. The fluid balance and critical illness are significant (*p* value < 0.05).

**Table 3:** Characteristics and clinical features of overall 294 COVID-19 infected patients.

	All patients (*n* = 294)	Deceased patients (*n* = 101)	Survivor patients (*n* = 193)	*p* ^*∗*^
Age, mean (SD) (y)	64.33 (16.38)	66.77 (16.56)	63.01 (16.21)	0.064

*Sex*				
Male	152 (51.71)	66 (65.34)	86 (44.55)	0.001
Female	142 (48.29)	35 (34.66)	107 (55.45)	

*Coexisting conditions*				
Hypertension	159 (54.08)	50 (49.50)	109 (56.47)	0.255
Diabetes	80 (27.21)	33 (32.67)	47 (24.35)	0.128
Malignancy	16 (5.44)	8 (7.92)	8 (4.14)	0.175
Cardiovascular disease	61 (20.74)	19 (18.81)	42 (21.76)	0.554
Chronic kidney disease	16 (5.44)	4 (3.96)	12 (6.21)	0.418
Respiratory disease	20 (6.80)	4 (3.96)	16 (8.29)	0.161
Heart disease	196 (66.66)	67 (66.33)	129 (66.83)	0.931
Heart failure	61 (20.74)	23 (22.77)	38 (19.68)	0.536
Critical illness	187 (63.60)	91 (90.09)	96 (49.74)	<0.001

Interleukin-6 (pg/mL), median (IQR)	92.4 (35, 233.5)	120.4 (56.3, 260.2)	75.8 (21.6, 175.1)	0.020

Procalcitonin (ng/mL), median (IQR)	3.7 (1.1, 14.5)	5.5 (3.2, 18.9)	1.4 (1.2, 3.8)	0.003

*White blood cell count (×10* ^*9*^ */L)*				
1600–11000	183 (62.25)	62 (61.39)	121 (62.69)	0.826
11001–48400	111 (37.75)	39 (38.61)	72 (37.30)	

*C-reactive protein (mg/L)*				
0.5–10	37 (12.58)	7 (6.94)	30 (15.54)	0.034
10–116	257 (87.41)	94 (93.06)	163 (84.45)	

Fluid balance during hospitalization, mL, median (IQR)	−250 (−550, 240)	150 (−100, 350)	−390 (−570, 100)	<0.001

Creatinine, mg/dL, median (IQR)	2.6 (0.8, 4.1)	2.2 (1.1, 4.2)	2.4 (1.1, 4.1)	0.053

Creatinine^*∗*^, mg/dL, median (IQR)	2.4 (0.8, 3.8)	2 (1, 4.2)	2.2 (0.9, 4)	0.032

Change of creatinine, mg/dL, median (IQR)	0.2 (0.1, 0.3)	0.2 (0.1, 0.28)	0.2 (0.1, 0.3)	0.943

All laboratory values from the day of admission. ^*∗*^Before discharge from the hospital in survived patients and before death in deceased patients.

## Data Availability

The data supporting the findings of this study are available on request from the corresponding author and with permission from the Babol University of Medical Sciences, Babol, Iran.

## Abbreviations

ARDS: Acute respiratory distress syndrome
ARF: Acute renal failure
ICU: Intensive care unit
BNP: Brain natriuretic peptide
NT-proBNP: N-terminal-pro hormone BNP
RT-PCR: Reverse transcriptase-polymerase chain reaction
SARS-CoV-2: Severe acute respiratory syndrome coronavirus 2
LVEF: Left ventricular ejection fraction
eGFR: Estimated glomerular filtration rate
ACE-2: Angiotensin-converting enzyme II
TMPRSS2: Transmembrane protease serine 2
AKI: Acute kidney injury
IL: Interleukin
TNF: Tumor necrosis factor.
